# Prenatal Diagnosis of Joubert Syndrome 23 With Left Isomerism: A Novel Phenotype Associated With Pathogenic KIAA0586 Variant

**DOI:** 10.1002/pd.70008

**Published:** 2025-10-28

**Authors:** Tamara Casteleyn, Markus Vogt, Alexander Weichert, Béla Zimmer, Birgit Lala, Wolfgang Henrich, Josefine Theresia Königbauer

**Affiliations:** ^1^ Department of Obstetrics Charité University Hospital Berlin Germany; ^2^ Prenatal Medicine Bergmannstrasse Berlin Germany; ^3^ Medicover Diagnostics Martinsried Germany; ^4^ Department of Radiology Charité University Hospital Berlin Germany

**Keywords:** ciliopathy, joubert syndrome, KIAA0586, laterality defect, left isomerism, prenatal diagnosis, TALPID3

## Abstract

Joubert syndrome is a rare autosomal recessive ciliopathy defined by the “molar tooth” sign caused by cerebellar vermis hypoplasia and abnormal superior cerebellar peduncles. Over 40 genes are known to cause the disorder, including KIAA0586, which encodes the centrosomal protein TALPID3, essential for ciliogenesis and Hedgehog signaling. Although KIAA0586‐related Joubert syndrome is primarily neurodevelopmental, cilia also play a key role in left‐right axis formation, however, laterality defects have not been reported in this context. We describe a prenatal case with suspected Joubert syndrome and left isomerism carrying a homozygous likely pathogenic KIAA0586 frameshift variant. The fetus showed dextrocardia, azygos continuation of the inferior vena cava, median gallbladder, persistent right umbilical vein, and a suspected molar tooth sign. This case suggests a possible phenotypic expansion of KIAA0586‐related ciliopathy and supports a role for TALPID3 in human left‐right patterning.

## Bulleted Statement

- What is already known about this topic?
  ◦ Joubert syndrome is genetically heterogeneous, with over 40 associated genes. *KIAA0586* mutations cause Joubert syndrome 23, a ciliopathy primarily affecting neurodevelopment, without reported laterality defects.
- What does this study add?
  ◦ To our knowledge, this is the first reported prenatal patient with *KIAA0586*‐related Joubert syndrome with left isomerism, suggesting a potential expansion of the phenotypic spectrum and a role of TALPID3 in human left‐right axis development.

## 1 Introduction

Joubert syndrome is a rare autosomal recessive ciliopathy characterized by the molar tooth sign, resulting from cerebellar vermis hypoplasia and abnormal superior cerebellar peduncles [1]. It is genetically heterogeneous, with over 40 associated genes [1] including KIAA0586, which encodes the centrosomal protein TALPID3, essential for primary ciliogenesis and Hedgehog signaling [2, 3].

While Joubert syndrome is primarily a neurodevelopmental disorder, cilia play a key role in embryonic left‐right axis formation, and laterality defects have been described in a subset of ciliopathies [3].

However, isomerism has not been reported for KIAA0586 variants. We present a prenatal patient with suspected Joubert syndrome and left isomerism, carrying a likely pathogenic frameshift variant in KIAA0586, suggesting possible phenotypic expansion.

## 2 Case Presentation

**FIGURE 1:**
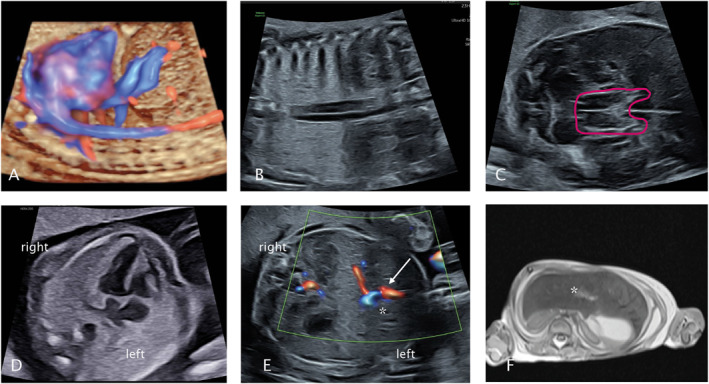
Sonographic images of the fetus in 22 gestational weeks showing azygos continuation of the inferior vena cava (A + B), molar tooth sign (C) and left isomerism with dextrocardia (D), a median located gallbladder (*) and persistent right umbilical vein (↑, E). The post mortem MRI (F) confirmed left isomerism with a horizontally oriented liver (*) and showed gallbladder aplasia and a hypoplastic spleen.

A 30‐year‐old healthy gravida 2, para 1 was referred at 12 + 6 weeks of gestation for first‐trimester screening. Her husband is her first cousin and their previous child is healthy. Fetal echocardiography revealed isolated dextrocardia (HP:0001651) with normal cardiac anatomy (Table 1A, Figure 1D). The non‐invasive prenatal test was unremarkable.

**TABLE 1A:** Clinical findings.

Case	Parental details			Gestation at diagnosis	Phenotypes (HPO terms)	Obstetric history	Family history	Outcome
1	Maternal	Age	30	12 + 6 weeks dextrocardia 19 + 5 weeks joubert syndrome	Dextrocardia (HP:0001651) Left isomerism (HP:0031854) Azygos continuation of the inferior vena cava (HP:0011671) molar tooth sign (HP:0002419)	2G1P	Unremarkable, Parents are consanguineous (first cousins)	Termination of pregnancy 22 + 5 weeks
		Ethnicity	Iranian					
	Paternal	Age	34					
		Ethnicity	Iranian					

After genetic counseling, amniocentesis was performed at 16 + 6 weeks.

DNA was extracted from uncultured amniocytes. Exome sequencing was carried out on DNA from the fetus and biological parents (trio analysis). Target enrichment was performed using the Twist Human Core Exome Kit with the RefSeq Panel (36.6 Mb), and sequencing was conducted on the Illumina NovaSeq platform.

Sequence reads were aligned to the GRCh38/hg38 human reference genome. Variant calling for SNVs and CNVs was performed using the Illumina DRAGEN Bio‐IT platform. Variant annotation, prioritization, and classification were conducted using the Franklin by Genoox platform. The filtering strategy included exclusion of common variants (allele frequency > 1%) and focus on rare protein‐altering variants. Coverage of ≥ 20 × was achieved in 97.62% of the target regions.

A homozygous frameshift variant in *KIAA0586* was identified. It affects clinically relevant transcripts and likely leads to a premature stop codon near the N‐terminus. While reinitiation from an alternative downstream methionine is theoretically possible, a loss of function is consistent with the known disease mechanism. The variant is absent in the homozygous state from gnomAD but listed in ClinVar and previously reported in three siblings with Joubert syndrome [4] as well as one individual with ataxia [5]. It was classified as likely pathogenic according to the ACMG criteria (Table 1B). Both parents were confirmed to be heterozygous carriers and are consanguineous (first cousins).

**TABLE 1B:** Genetic findings.

Procedure (gest age)	Direct/culture?	Performed test	Secondary confirmatory test	Gene (name; REFSEQ)	Known disease (OMIM)	Variant	ACMG classify‐cation	Criteria applied	Inheritance & zygosity	Interpret‐ation
Amnio‐centesis 19 + 5 weeks	Direct	Whole exome sequencing	/	KIAA0586 NM_001329943.3	Joubert syndrome 23 (OMIM #616490)	Allele 1: c.38del; p.(Lys13Argfs*6)	Likely patho‐genic	PVS1_ StrongPM3_ moderate PM2_ supporting	Autosomal recessive, homozygous	Consistent with diagnosis
						Allele 2: c.38del; p.(Lys13Argfs*6)				

No pathogenic variants were found in known heterotaxy genes. Limitations of exome sequencing (e.g., non‐coding regions) apply.

After genetic counseling and psychosomatic support, the parents opted for termination of pregnancy at 22 + 5 weeks. The follow‐up ultrasound confirmed left isomerism (HP:0031854) with azygos continuation of the inferior vena cava (HP:0011671, Figure 1A–B, 1D), median gallbladder, persistent right umbilical vein (Figure 1E), and suspected molar tooth sign (HP:0002419, Figure 1C).

After performing a fetocide, labor was induced. The postmortem MRI confirmed left isomerism with a horizontally oriented liver, hypoplastic spleen, gallbladder aplasia, and dextrocardia (Figure 1F). Unfortunately, the calvaria was deformed, which made it impossible to assess the mesencephalon.

## 3 Discussion

KIAA0586 encodes TALPID3, a protein essential for ciliogenesis and Hedgehog signaling [3]. Although biallelic KIAA0586 variants cause Joubert syndrome [2], laterality defects have not been described in humans. Although the diagnosis of Joubert syndrome could not be confirmed postnatally as the calvaria was deformed, no other likely pathogenic variants, including in known heterotaxy genes, were identified by exome sequencing. Notably, KIAA0586 knockout mice show randomized cardiac laterality, supporting a link between KIAA0586 variants and laterality defects in this fetus [6].

While a single case cannot definitively establish causality, this case suggests a potential phenotypic expansion of KIAA0586‐related ciliopathy that warrants further investigation.

## Funding

The authors have nothing to report.

## Ethics Statement

Exome sequencing was performed as a clinical service under clinical consent forms. Retrospective collection of data from patient records has been granted a waiver of informed consent as all clinical data contained in this report have been anonymized. Ethics approval statement is not applicable.

## Consent

Written informed consent was obtained from the involved patient.

## Conflicts of Interest

The authors declare no conflicts of interest.

## Data Availability

The data that support the findings of this study are available on request from the corresponding author. The data are not publicly available due to privacy or ethical restrictions.

## References

[1] S. Gana , V. Serpieri , and E. M. Valente , “Genotype‐Phenotype Correlates in Joubert Syndrome: A Review,” American Journal of Medical Genetics Part C: Seminars in Medical Genetics 190, no. 1 (March 2022): 72–88, 10.1002/ajmg.c.31963. 35238134 PMC9314610

[2] R. Bachmann‐Gagescu , I. G. Phelps , J. C. Dempsey , et al., “ *KIAA0586* is Mutated in Joubert Syndrome,” Human Mutation 36, no. 9 (2015): 831–835, 10.1002/humu.22821. 26096313 PMC4537327

[3] A. M. Fraser and M. G. Davey , “TALPID3 in Joubert Syndrome and Related Ciliopathy Disorders,” Current Opinion in Genetics & Development 56 (2019): 41–48, 10.1016/j.gde.2019.06.010. 31326647

[4] S. Roosing , M. Hofree , S. Kim , et al., “Functional Genome‐Wide siRNA Screen Identifies KIAA0586 as Mutated in Joubert Syndrome,” eLife 4 (2015): e06602, 10.7554/elife.06602. 26026149 PMC4477441

[5] S. Hiz Kurul , Y. Oktay , A. Töpf , et al., “High Diagnostic Rate of Trio Exome Sequencing in Consanguineous Families With Neurogenetic Diseases,” Brain 145, no. 4 (2022): 1507–1518, 10.1093/brain/awab395. 34791078 PMC9128813

[6] F. Bangs , N. Antonio , P. Thongnuek , et al., “Generation of Mice With Functional Inactivation of Talpid3, a Gene First Identified in Chicken,” Development 138, no. 15 (2011): 3261–3272, 10.1242/dev.063602. 21750036 PMC3133916

